# A systematic study of arsenic adsorption and removal from aqueous environments using novel graphene oxide functionalized UiO-66-NDC nanocomposites

**DOI:** 10.1038/s41598-022-18959-2

**Published:** 2022-09-22

**Authors:** Simranjeet Singh, T. S. Sunil Kumar Naik, Basavaraju U, Nadeem A. Khan, Abdul Basit Wani, Sushant Kumar Behera, Bidisha Nath, Shipra Bhati, Joginder Singh, Praveen C. Ramamurthy

**Affiliations:** 1 Interdisciplinary Centre for Water Research (ICWaR), Indian Institute of Science, Bangalore, India; 2 Department of Materials Engineering, Indian Institute of Science, Bangalore, India; 3 Civil Engineering Department, Mewat Engineering College, Nuh, Haryana India; 4 Department of Chemistry, University of Kashmir, Srinagar, India; 5 Interdisciplinary Centre for Energy Research, Indian Institute of Science, Bangalore, India; 6 Department of Chemistry, The Oxford College of Engineering, Bangalore, India; 7 Department of Microbiology, Lovely Professional University, Phagwara, Punjab India

**Keywords:** Environmental sciences, Engineering

## Abstract

This study investigates the removal of As(V) from aqueous media using water stable UiO-66-NDC/GO prepared via the solvothermal procedure. The synthesized material was analyzed by Raman spectroscopy, UV–visible, X-ray powder diffraction (XRD), Transmission electron microscopy (TEM), Fourier Transform Infrared spectroscopy (ATR-FTIR), scanning electron microscopy (SEM), and Brunauer–Emmett–Teller (BET) support its applicability as a super-adsorbent for the adsorption of As(V) ions from aqueous solutions. The effect of various parameters, including initial ion concentration, temperature, adsorbent dose, and pH on the adsorption of As(V) was studied to recognize the optimum adsorption conditions. The q_max_ obtained for this study using Langmuir isotherms was found at 147.06 mg/g at room temperature. Thermodynamic parameters ΔH°, ΔG°, and ΔS° were also calculated and negative values of ΔG° represent that the As(V) adsorption process occurred exothermically and spontaneously. Meanwhile, theoretical density functional simulation findings are accommodated to support these experimental results. It is observed that the dynamic nature of graphene oxide and the UiO-66 NDC nanocomposite system becomes superior for adsorption studies due to delocalized surface states. UiO-66-NDC/GO also showed high reusability for up four regeneration performances using 0.01 M HCl as a regenerant.

## Introduction

Groundwater pollution is currently a major environmental issue all over the world, and it is frequently caused by the presence of different wastewater contaminants^1,2^. Arsenic (As) is one of the world's top 20 hazardous chemicals and it can be found in a variety of inorganic and organic forms. Combustion of fossil fuels, Mining, and insecticides are all examples of anthropogenic and natural sources of As pollution^3^. The World Health Organization (WHO) and Environmental Protection Agency United States (EPA) both recommended a 10-ppb threshold for drinking water^4^. Arsenic in inorganic forms (arsenate and arsenite), is more toxic than arsenic in organic forms and is found naturally in groundwater and soil^5^. Arsenic in inorganic forms affects more than 200 million population worldwide, and its long-term exposure causes serious illness, dysfunction of the nervous system, skin cancer, lung cancer, kidney failure, liver diseases, urinary bladder cancer, cardiovascular and peripheral disease^6^. Large-scale groundwater poisoning by As in Bangladesh in the 1990s was the world's largest poisoning occurrence^7^. The health of nearly 100 million Indians is threatened by groundwater contamination of As^8^. As a result, arsenic contamination is a severe issue that requires the development of effective clean-up technologies.

Various treatment techniques have been documented for arsenic removal from water, including adsorption, bioremediation, coagulation-flocculation, ion exchange, electrochemistry, sedimentation, precipitation, membrane filtration, reverse osmosis, normal filtration, and lime softening^9^. From the above-stated methods, the adsorption process has been reported most regarding the eradication of arsenic because of its flexibility in process design, cost-effectiveness, and operational simplicity. To date, different adsorbents have been developed by researchers, including activated carbon, titanium oxide, activated alumina, zirconium oxide, iron oxide, Fe (III) loaded resins, iron oxide, metal oxides, agricultural biomasses, goethite, zerovalent iron, mesoporous alumina, different metal-based nanocomposites for removal of As from contaminated aquatic bodies^10–12^. All these materials are efficient, and their application on a large scale is restricted due to high operational cost, low adsorption potential, and extended time of consumption. Therefore, there is a continuous demand to synthesize novel and efficient adsorbents with improved adsorption capacity for arsenic decontamination from water.

A new hybrid material, commonly known as Metal–organic frameworks (MOFs), built from inorganic metal and the organic linker, has gained large attraction over the last few years. Owing to their customizable chemical functionalities, good thermal stability, tunable pore size, and versatile architectures, having broader applications in sensing, gas storage, catalysis, wastewater treatment, separation, etc. In recent years, Zr-MOF-based composites have potentially been applied for the As (III) and As(V) removal from water due to their excellent adsorption capacity. Guo et al. synthesized a composite membrane Zr-based MOF (UiO-66) and polyacrylonitrile (PAN) for efficient removal of both arsenite (AsIII) and arsenate (AsV) from water^13^. As a result, in most arsenic removal procedures, oxidation of arsenate to arsenite occurs as a pre-treatment phase^14^.

Arsenic removal studies were carried out on different MOFs such as Fe-BTC MOF, MIL-53, and ZIF-8. However, no noteworthy results were found compared to other synthetic commercial and adsorbents. Therefore, developing and exploring new MOF nano adsorbents having high adsorption capacities are particularly interesting. In recent years, Zr-MOF-based composites have potentially been applied for the removal of As(V) from water due to their excellent adsorption capacity. For the adsorption of Arsenic (V) heavy metal from an aqueous solution, a new nano adsorbent material was synthesized in this research work by employing graphene oxide (GO) and a zirconium-based metal–organic framework. The UiO-66-NDC is a three-dimensional structure of one octahedral centre hole cage and eight tetrahedral corner cages of secondary building units Zr_6_O_4_(OH)_4_ and twelve bridge ligands 1,4-NDC. It has a significant number of coordinatively unsaturated Zr^4+^ sites and a strong ZrO bond, which aids in adsorption and mass transfer. These materials are resistant to hydroxide ions and protons and are stable in a variety of wastewater types and pH levels.

In the present study, a novel nanocomposite adsorbent material was synthesized using graphene oxide (GO) and a zirconium-based metal–organic framework, i.e., UiO-66-NDC [Zr_6_O_4_(OH)_4_(1,4-NDC)_6_]n and used as an adsorbent to uptake aquatic arsenate As(V). The UiO-66 framework consists of Zr_6_O_4_(OH)_4_ clusters and 1,4-NDC, i.e., 1,4-naphthalene dicarboxylate linker. The As(V) adsorption capacity was examined by detailed characterizations and adsorption mechanism studies. This research reveals the superior performance of the MOF-based nano-adsorbent in the removal of arsenic from water, which could lead to new insights into the use of MOFs in water treatment and the development of an enhanced adsorbent material for the arsenic decontamination sector.

## Results and discussion

### Simulation output

Geometry optimized state of the GO and nanocomposite systems have been obtained via Broyden-Fletcher-Goldfarb-Shanno (BFGS) algorithm^15–17^. It is obvious from the total energy values that nanocomposite system (− 839.231 Ry) achieves dynamic superiority over the GO system (− 179.291 Ry) because of the Zr and Cl atoms present in the system. As a result, this dynamic stability of the UiO-66-NDC/GO nanocomposite makes a relevant platform to adsorb arsenic (As) like metalloids metals and can be used as the perfect candidate for adsorption studies. Eventually, a pristine GO system has only localized states of a carbon atom, which gives less total energy without any dynamic nature for adsorption. Similarly, Zr based delocalized surface states are coming into the picture in the UiO-66-NDC/GO nanocomposite system, compared to the localized surface states of carbon in the GO system. Generally, surface states are states on the surface, and their electronic wavefunction can be localized (i.e., trapped) or delocalized (i.e., conductive or in-motion). If the surface states are caused by composite formation, then it is most likely that the resulting surface states are delocalized. However, all functional parameters strongly depend on surface states and their relaxation. In another case, surface states caused by surface defects or some passivating molecules are, most likely, localized surface states. This phase transformation from the localized state in GO to delocalized states in nanocomposite makes the nanocomposite system a relevant surface to adsorb As metalloids. It makes the nanocomposite surface adsorption active for As adsorption and removal process supporting the aforementioned experimental results strongly.

### UV–Vis results

**Figure 1:**
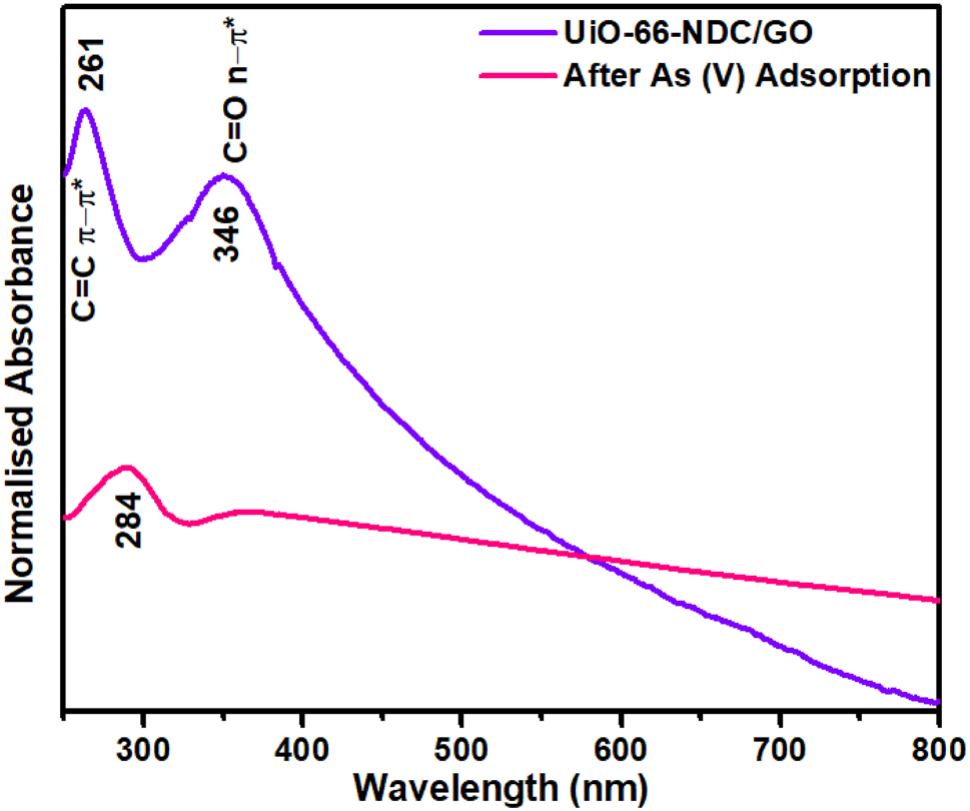
UV–Vis analysis of UiO-66-NDC/GO nanocomposite before and after experiment.

In UV–Vis spectra of UiO-66-NDC/GO nanocomposite, there are two characteristic absorption peaks with maxima at 261 and 346 nm (Fig. 1). The absorption centred at 346 nm is because of the π–π* transition of naphthalene rings^18^, whereas absorption at 261 nm is shifted π–π* transition of graphene oxide in UiO-66-NDC/GO nanocomposite^19^. There is a prominent change in the absorption spectrum of nanocomposite after As(V) uptake. The absorption peak at 346 nm completely disappeared while there was a redshift (with hypochromic effect) to the π–π* transition of naphthalene rings to 284 nm. These notable alterations suggest the binding of As(V) on UiO-66-NDC/GO nanocomposite.

### FTIR studies before and after As(V) adsorption

**Figure 2:**
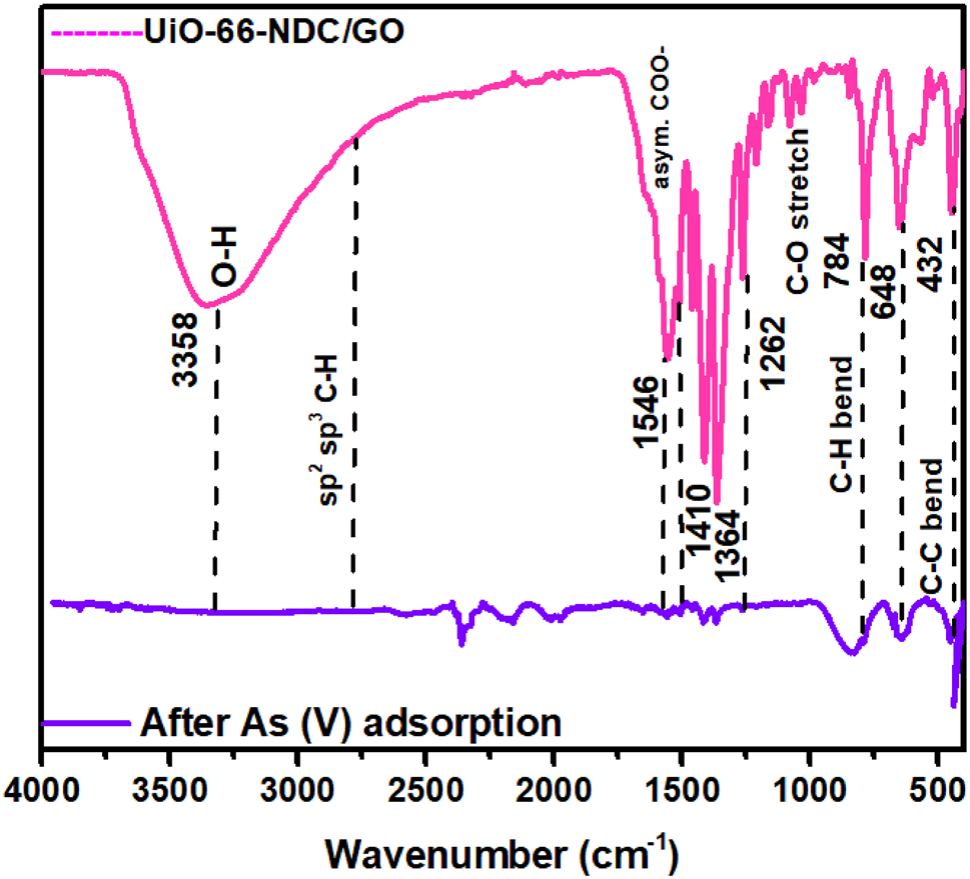
FTIR spectrum of UiO-66-NDC/GO nanocomposite before and after experiment.

The functional characterization of UiO-66-NDC/GO was carried out using FTIR spectroscopy in ATR mode. The FTIR spectrum of UiO-66-NDC/GO was recorded before and after As(V) adsorption. The peak at 3355 cm^−1^, which is attributed to the O–H stretching vibration of the carboxylic acid group, completely disappeared after As(V) adsorption. The asymmetric stretch at 1546 cm^−1^ and symmetric stretch at 1364 cm^−1^ are attributed to COO^−^ groups of UiO-66-NDC/GO nanocomposite^20^. After As(V) adsorption, both of these antisymmetric and symmetric stretching vibrations are shifted with a prominent decrease in intensity. C-H bending vibration at 784 cm^−1^ and C=C bending vibration at 648 cm^−1^ got shifted by adsorption of As^21^. In other groups, such as C=O and C–O group related to carboxyl, no significant change was observed after arsenate adsorption; only the intensity of the peak was found to be decreased. These results imply the efficiency of O–H and aromatic C=C groups in arsenate adsorption. The prominent shifts in the FTIR spectrum after adsorption of As(V) establish the robust interaction between the nanocomposite and As(V) (Fig. 2).

### Raman studies

**Figure 3:**
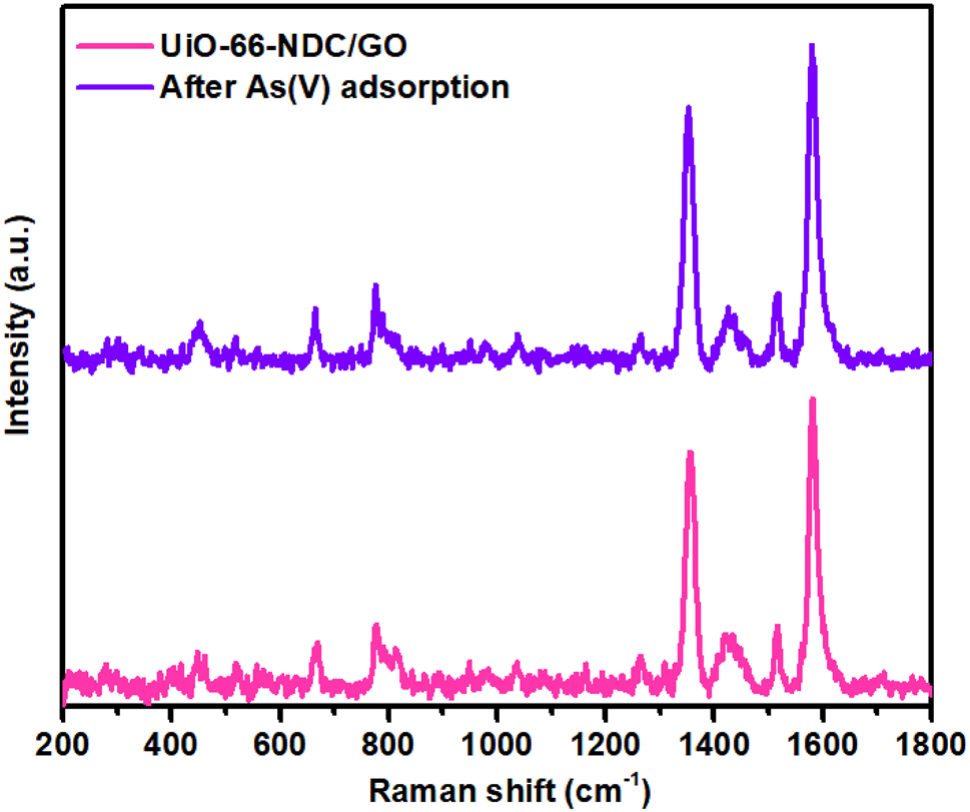
Raman data of as synthesized and As(V) adsorbed UiO-66-NDC/GO nanocomposite.

After that, Raman measurements were carried out to understand the structural variation of UiO-66-NDC/GO. Raman data was collected as synthesized GO/ UiO-66-NDC and As(V) adsorbed UiO-66-NDC/GO. Raman vibrational peaks at 1580 cm^−1^ and 663 cm^−1^ are related to C=C stretching and in-plane bending vibrational frequency of aromatic ring of NDC linker. Raman vibrational peaks at 1354 cm^−1^ and 1516 cm^−1^ were related to in-plane ring deformation band of NDC linker. The Raman doublet peaks in the range of 1400 – 1500 cm^−1^ are related to the in-plane symmetric stretching vibrational band of O=C–O group in NDC linker. The Raman peak at 773 cm^−1^ was related to C–H in-phase wagging band of NDC linker. However, we could not resolve Raman bands related to GO owing to a strong and competitive peak position of NDC linker^22^. The Raman vibrational bands in synthesized material are well matched with previously reported data of UiO-66-NDC. After As(V) adsorption, there are no significant changes in Raman peak position and FWHM of UiO-66-NDC/GO. It means that Raman vibrational bands of GO/UiO-66-NDC are not influenced by the As(V) adsorption (Fig. 3).

### XRD studies

**Figure 4:**
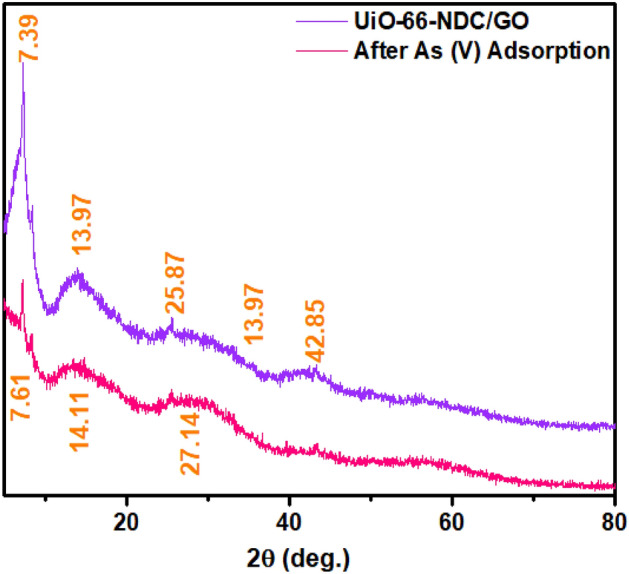
XRD spectra analysis of GO/UiO-66-NDC nanocomposite before and after experiment.

To understand the structural changes during the adsorption process, XRD analyses are carried out for UiO-66-NDC/GO nanocomposite. The diffraction lines at 2θ of 7.39° and 8.4° are represented characteristic peaks of UiO-66. Hence, the synthesized UiO-66-NDC has the same crystalline structure. These diffraction line intensities are drastically decreased after the adsorption of As(V), which says that reduction in the crystalline nature of UiO-66-NDC/GO, is shown in Fig. 4. The amorphous nature complements the adsorption properties providing defect sites for heavy metal adsorption. The sharp resonances near 7 deg. is characteristic of graphene oxide^23^.

### SEM characterization

**Figure 5:**
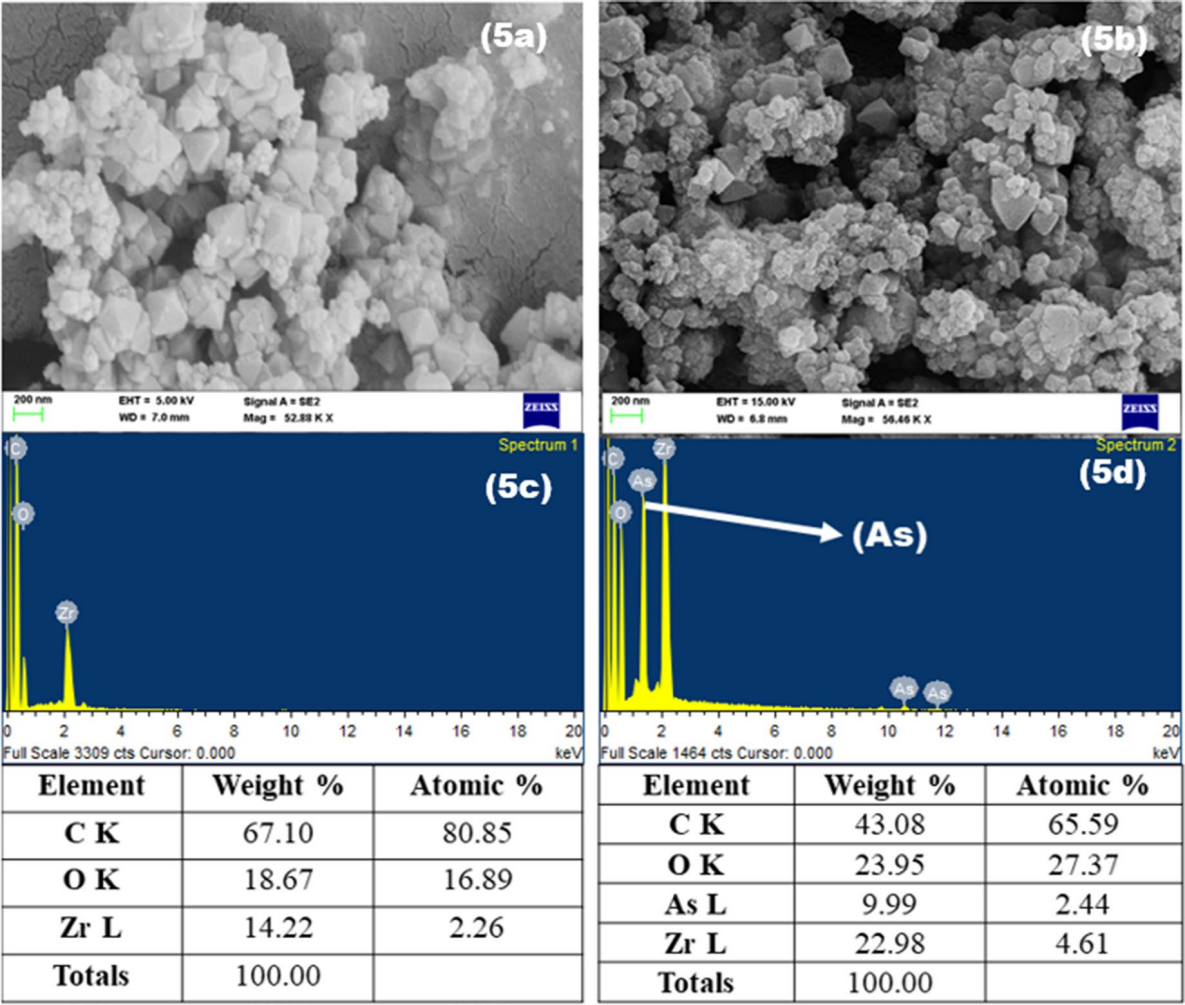
SEM micrograph analysis of UiO-66-NDC/GO nanocomposite before and after experiment.

In order to reveal the surface structure of the UiO-66-NDC/GO before and after As(V) adsorption, the SEM–EDS was performed. As represented in Fig. 5, changes were observed in the structure and morphology of the GO-UiO66-NDC nanocomposite after As(V) adsorption. After the adsorption studies, it is clearly notable that the As(V) was adsorbed on the surface of the GO-UiO66-NDC nanocomposite. From EDS data, we observed that there is no existence of As element in synthesized UiO-66-NDC/GO; however, in the other sample, 2.44 atomic% of As element is observed. This clearly indicates that the As(V) is adsorbed on the UiO-66-NDC/GO surface.

Based on the EDS observations of the UiO-66-NDC/GO nanocomposite, there are different elements, including carbon, zirconium, and oxygen (Fig. 4c). EDX analysis after As(V)adsorption clearly represents the presence of As species in the UiO-66-NDC/GO nanocomposite structure, which is likely to the adsorption As(V) (Fig. 4d). Furthermore, a decrease in the intensity of carbon peaks is evident in the EDX analysis of UiO-66-NDC/GO following the As(V) sorption process supporting the successful adsorption of As(V) by UiO-66-NDC/GO.

### TEM measurements

**Figure 6:**
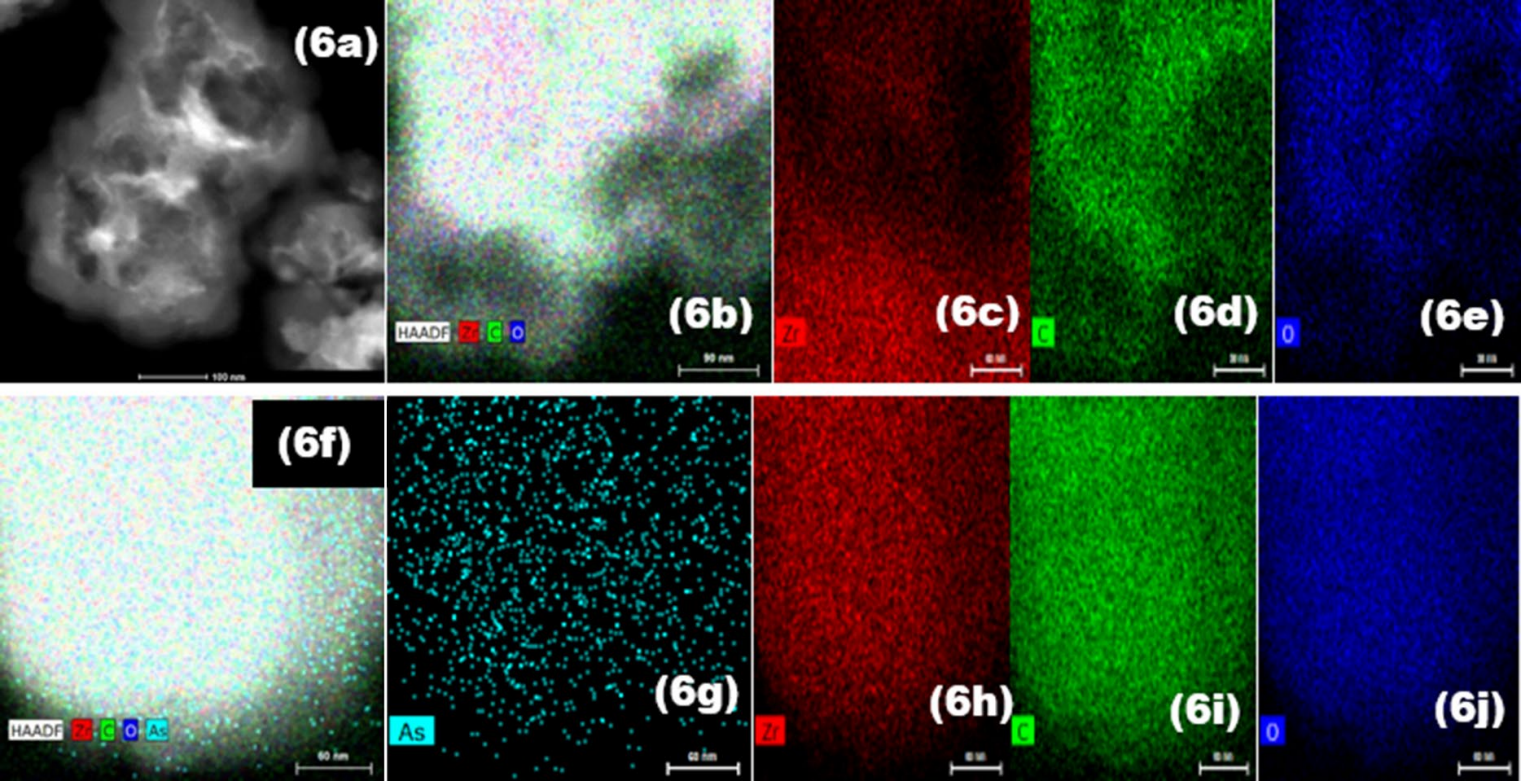
TEM analysis and mapping of UiO-66-NDC/GO before (**a**–**e**) and after the As(V) adsorption (**f**–**j**).

To study the chemical composition and surface morphology of the UiO-66-NDC/GO before and after the As(V) adsorption, TEM with EDS mapping was performed. The particles of UiO-66-NDC/GO are separated in some areas and agglomerated in others, as seen in Fig. 6a. The elemental analysis of the UiO-66-NDC/GO was also determined by TEM-EDS mapping. Before the experiment, bare UiO-66-NDC/GO showed an abundance of zirconium, carbon and oxygen (Fig. 6c–e) at the surface and after adsorption, traces of As (Fig. 6g) were also seen along with the pristine UiO-66-NDC/GO. As(V) adsorption on the surface of the UiO-66-NDC/GO may be the reason why the TEM structure of the UiO-66-NDC/GO showed a smoother surface after the As(V) was removed.

### BET measurements

**Figure 7:**
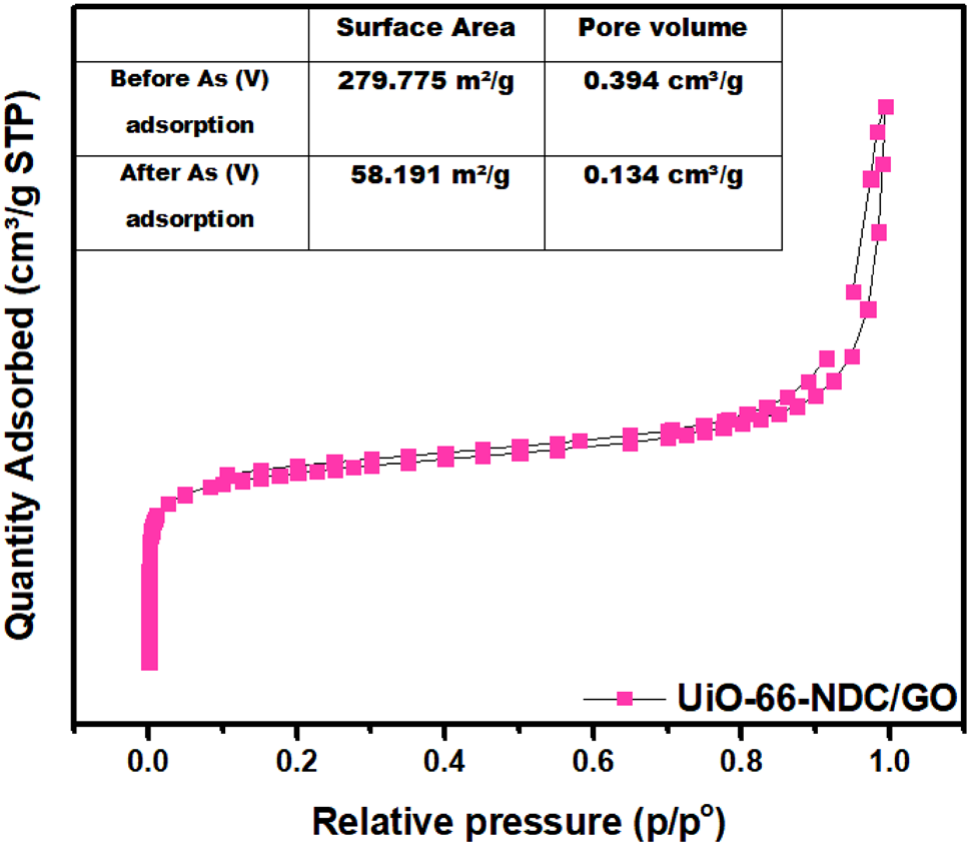
Pore size distribution and N_2_ sorption isotherms of UiO-66-NDC/GO before and after As(V) adsorption.

BET measurements were carried out before and after As(V) studies and the data is represented in Fig. 7. The volume of pore and surface area for the UiO-66-NDC/GO was determined as 0.394917 cm^3^/g and 279.7756 m^2^/g, respectively. After the adsorption experiment, the surface area and pore volume decreased to 58.1915 m^2^/g and 0.134 cm^3^/g. This decrease suggests that the As(V) species permeate into the pores of UiO-66-NDC/GO. The UiO-66-NDC/GO surface pores and comparably adequate active surface allow for fast transfer and adsorption of As(V) from an aqueous solution to the UiO-66-NDC/GO.

### Effect of the pH on As(V) adsorption

**Figure 8:**
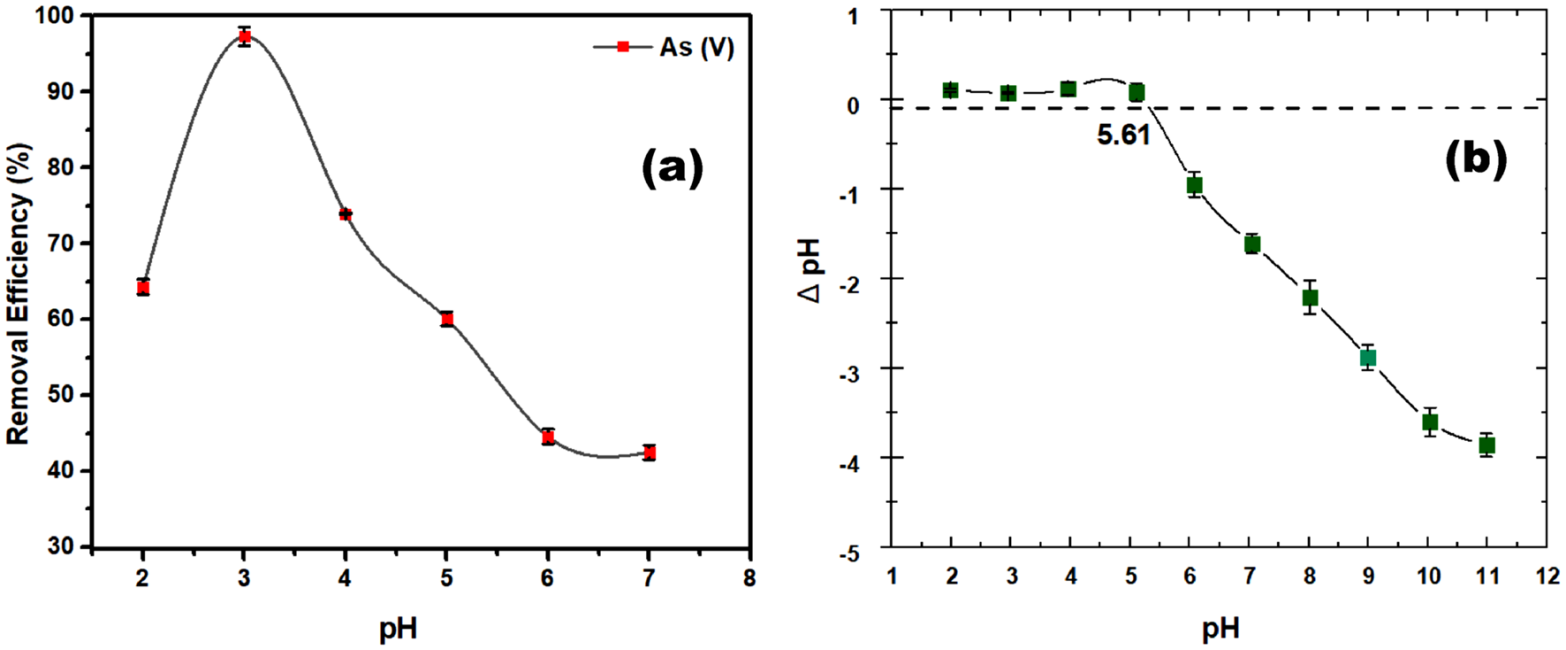
Effect of pH (**a**) and PZc (**b**) on As(V) removal and adsorption. pH = 2–7, T = 25 °C, and UiO-66-NDC/GO dose = 0.1 g/L, As(V) concentration = 5 mg/L.

The pH of the solution is an important parameter in arsenate adsorption studies since it influences arsenate speciation, distribution of contaminant species, and the adsorbent's surface charge. The pH effect on arsenate removal by UiO-66-NDC/GO adsorbent was examined at pH levels ranging from 2 to 7 and the results are shown in Fig. 8. The adsorption process for As(V) increased with an increment in pH from 2 to 3 and decreased dramatically with the increase in pH values from 4 to 7. The maximum removal capacity of 98.31% was observed at pH3 with 5 mg L^−1^ of initial As(V) concentration at an adsorbent dose of 0.1 g L^−1^. Further increasing the pH to 7; however, the arsenate removal efficiency decreased considerably to 42.5%. Based on the above observation, the possible removal mechanism can be explained as follows: The arsenate exists in various forms in water, such as: at pH below 2.0, it exists in H_3_AsO_4_, H_2_AsO_4_^−^ at pH from 2.0 to 3.0, and at pH 4.0–10.0 as HAsO4^2−^ respectively. The point of zero charges (pHpzc) was found to be 5.61, representing a positively charged outer surface of UiO-66-NDC/GO adsorbent at pH below 5.61 and at pH above 5.61 signifies a negatively charged outer surface. Therefore, when the pH is 3, negatively charged As(V) species (H_2_AsO_4_^−^) are attracted to the positively charged surface of the UiO-66-NDC/GO nanocomposite adsorbent. As(V) uptake increases due to electrostatic interaction between the sorbent and the H_2_AsO_4_^−^ ions, and the adsorption mechanism was through electrostatic attraction. However, below pH 3.0, a decreased adsorption capacity is observed due to the arsenate being present as H_3_AsO_4_ and a strong competition existed between H_3_AsO_4_ and protons for adsorption sites. At higher pH, i.e., between pH 4.0–7.0, excessive OH^−^ ions compete with predominant species of arsenate, i.e., HAsO4^2−^ ions, for adsorption on the adsorbent surface, of which OH^−^ ions dominate.

Therefore, the adsorption capacity of As(VI) decreases with an increase in the pH. The adsorption mechanism of As(V) was mainly coordination and ion exchange with metal nodes of the composite material. The influence of electrostatic forces at pH 3 drew anionic arsenate species to the vicinity of positively charged adsorbents, resulting in improved adsorption performance. The highest removal performance was obtained at pH 3 due to electrostatic force and acid–base interaction between adsorbent and arsenic species. At high acidic conditions (pH 2), H_3_AsO_4_ releases H ions and is attached to the hydroxyl sites in UiO-66-NDC/GO facilitating the arsenic uptake process.

### Effect of the UiO-66-NDC/GO dose on As(V) adsorption

**Figure 9:**
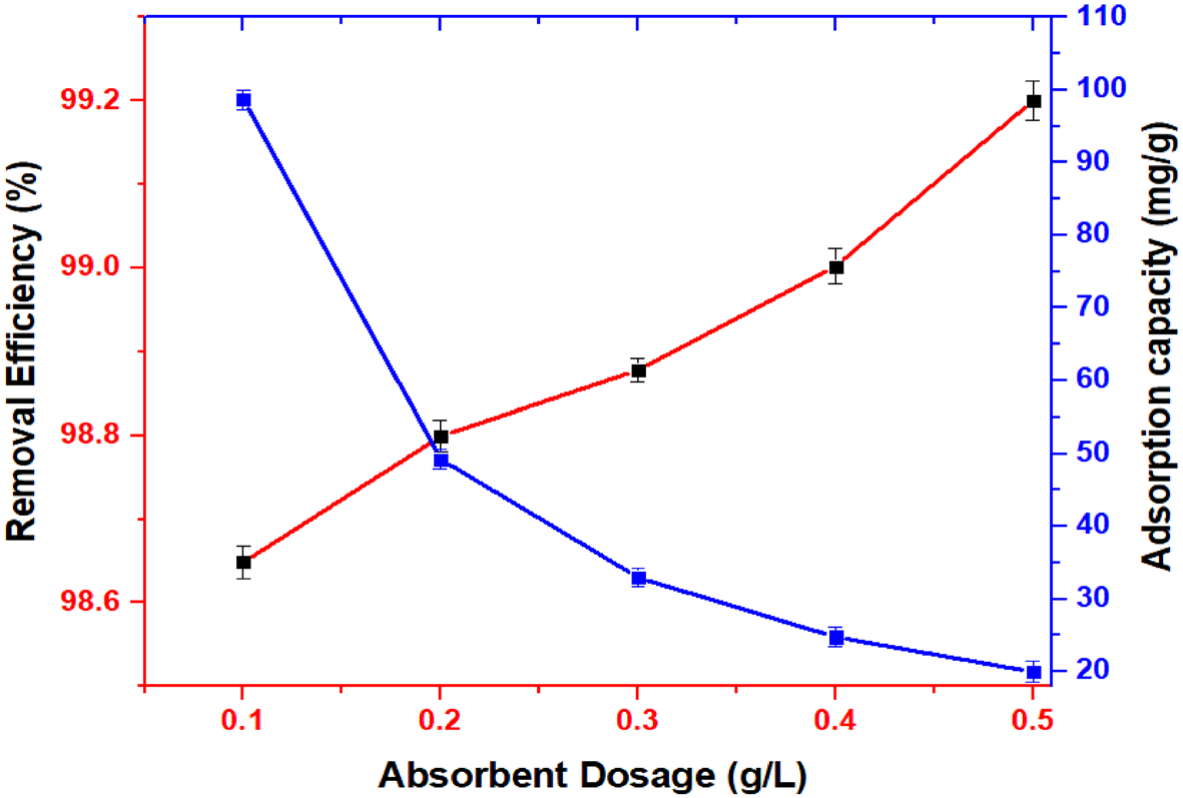
Effect of dose on As(V) removal and adsorption. pH = 3, T = 25 °C, and UiO-66-NDC/GO dose = 0.5–1.5 g/L, As(V) concentration = 5 mg/L.

The effect of adsorbent concentration on As(V) removal is illustrated in Fig. 9. The adsorbent was highly effective and quickly removed As(V) from water. The removal percentage was more than 98.67% at a low concentration of adsorbent dosages. The adsorption capacity decreased slightly as the dosage of the adsorbent increased from 0.1 to 0.5 g. As the adsorption period increases, the remaining concentration of As(V) in the solution decreases, lowering the static driving force of mass transfer. As a result, adsorption is rapid at first, while the concentration gradient is high and slows after 30 min as the system approaches equilibrium. This could be due to the accumulation of MOFs and the sorbent's reduced effective active site for pollutant removal^24^. As a result, the best adsorbent dose for As(V) sorption was determined to be 0.1 g/L. At this dose, the adsorption efficiency for As(V) was 98.67%, respectively.

### Effect of the initial concentration on As(V) adsorption

**Figure 10:**
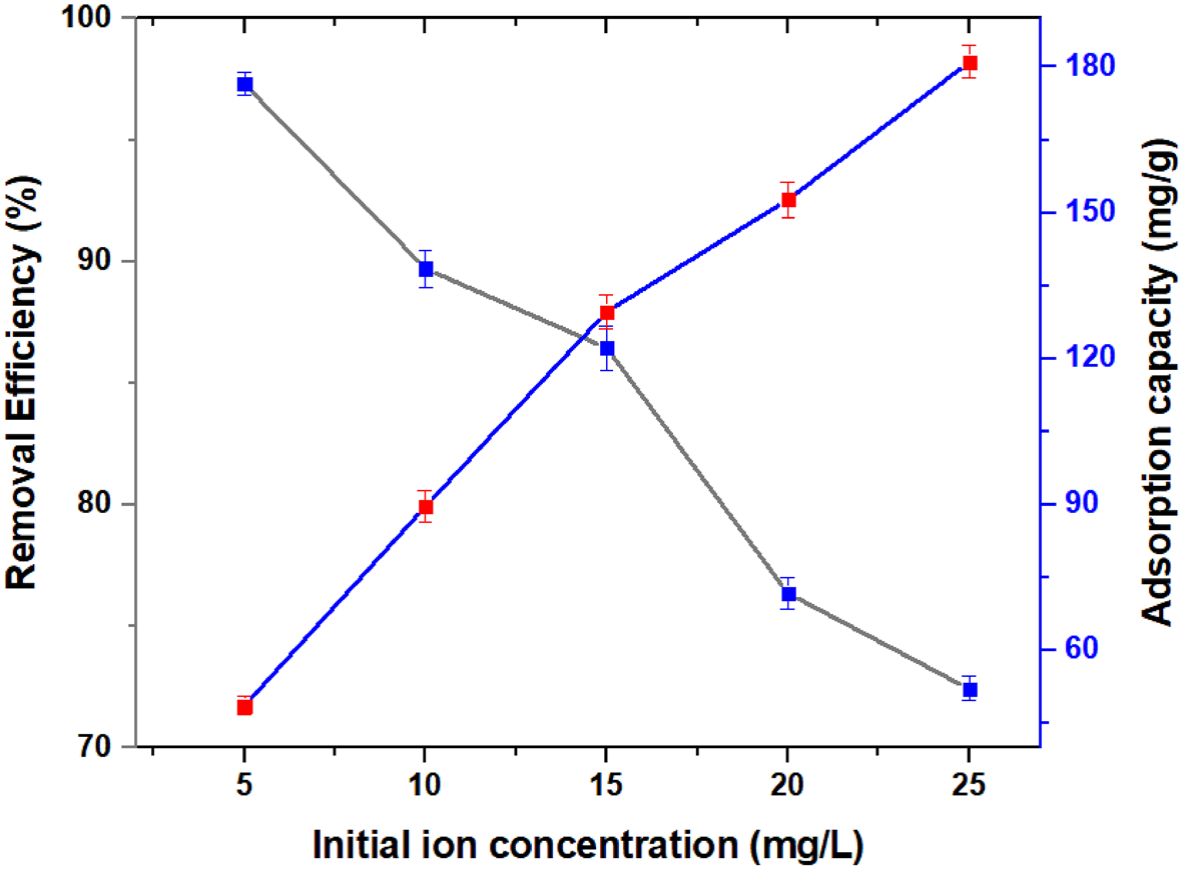
Effect of initial ion concentration on As(V) removal and adsorption. pH = 3, T = 25 °C, and UiO-66-NDC/GO dose = 0.5 g/L, As(V) concentration = 5–25 mg/L.

Changes in As(V) initial concentrations clearly impacted As(V) removal efficiencies. The removal percentage of As(V) declined significantly as the initial As(V) concentration increased in 30 min, but these rates were near all the same after 30 min. As the initial concentration of As(V) increased from 5 to 25 mg/L, the adsorption capacity increased from 48.64 to 181.05 mg/g of the nanocomposite (Fig. 10), explaining that adsorbent had a high adsorption capacity to As(V) at different initial concentrations. This increased adsorption capacity could be owing to a higher mass transfer driving force between the As(V) solution and the adsorbent, as well as a higher possibility of As(V) colliding with MOF nanocomposite. In addition to the previously described factors, the increase in adsorption capacity with increasing initial As(V) concentrations can be attributed to the driving force created in response to the concentration gradient, as well as strong interactions between the As(V) species and the MOF adsorbent.

### Adsorption kinetics

Six kinetic models (pseudo-first-order and five different types of Pseudo second order) were used to investigate the adsorption behaviour of adsorption processes whose limiting step may entail chemical reactions, surface adsorption, or diffusion transport. Pseudo-order kinetic models (1 and 2) are the most frequent models used to study adsorption kinetics. In addition to this, on linear Elovich model was also used to study the chemical nature of adsorption behaviour of UiO-66-NDC/GO on to As(V) ions in Eq. (3).

**Figure 11:**
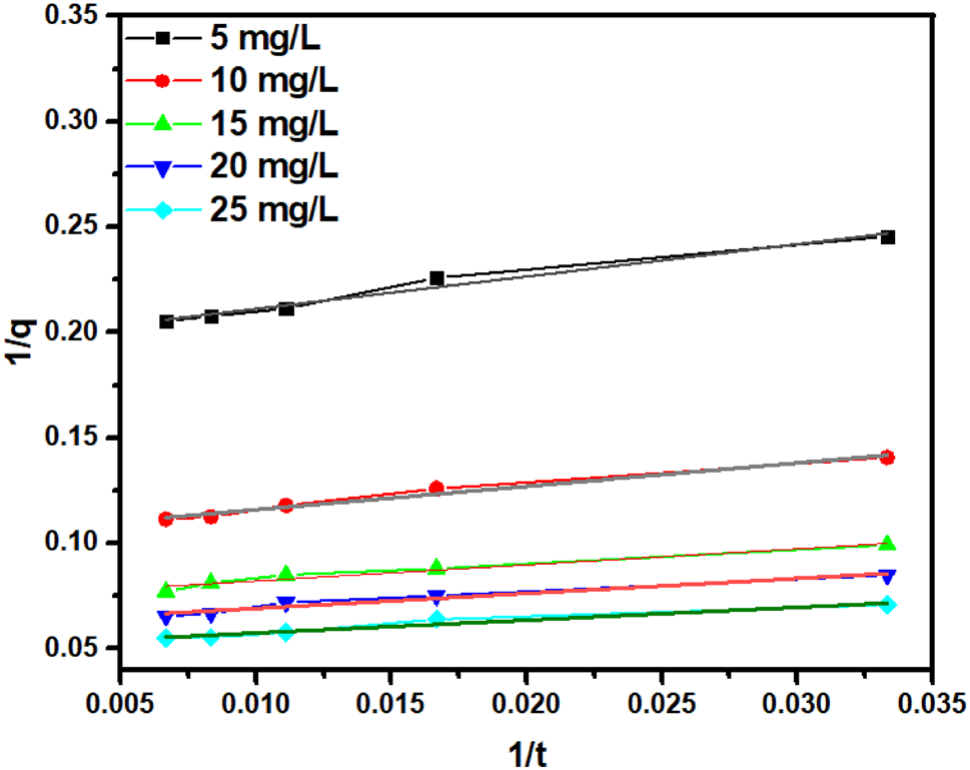
As(V) adsorption kinetics of UiO-66-NDC/GO as determined by PSO adsorption kinetics (Type 2).

According to a pseudo-kinetic (first-order) model, the change in the rate concentration solute with time, as well as changes in concentration of adsorbate and adsorbent dose over time (1), are logarithmically proportional. According to a pseudo-second-order kinetic model, the number of active sites occupied on the material is directly proportional to the adsorption capacity (2).

1 $$\mathrm{log}\left({q}_{e}-q\right)= \mathrm{log}\left({q}_{e}\right)-\frac{{K}_{1}t}{2.303}$$

2 $$\frac{t}{q}=\frac{1}{{K}_{2}{q}_{e}^{2}}+\frac{1}{{q}_{e}}t$$

3 $$qt= \frac{1}{\beta }\mathrm{ln}(\alpha \beta t +1)$$

where q_e_ = equilibrium adsorption capacity (mg/g),α = initial adsorption rate, β = desorption constant, q_t_ adsorbed amount at a time (mg/g) and k_1_ and k_2_are rate constants of pseudo order first and second respectively. The data obtained from the kinetic model are represented in Fig. 11. Table 1 illustrates the data obtained from kinetic parameters according to the value of the correlation coefficient.

**Table 1:** Kinetic parameters of As(V) adsorption on UiO-66-NDC/GO determined from best fits to PSO (Type 2) model using linearized fits.

Parameters	Pseudo-second-order kinetic model (type 2)				
y = mx + c	y = 1.53x + 0.19	y = 1.10x + 0.10	y = 0.76x + 0.07	y = 0.70x + 0.06	y = 0.60x + 0.05
Intercept	0.19591	0.1049	0.0745	0.0620	0.05138
Slope	1.5322	1.1070	0.7631	0.7093	0.6092
Experimental	4.86	8.97	12.96	15.27	18.10
Theoretical	5.10	9.53	13.42	16.10	19.45
R^2^	0.97	0.98	0.95	0.96	0.95

The kinetics of As(V) adsorption on UiO-66/GO followed the pseudo-second-order model type 2, as shown by the correlation coefficient (R^2^). The theoretical values calculated for type 2 pseudo order kinetics are almost similar to the experimental one representing the chemisorption mechanism. Although type 2 PSO can determine whether overall adsorption rate but can’t predict the diffusion process during adsorption. Furthermore, the results obtained from the pHpzc studies and adsorption kinetics studies of the composites demonstrated the effectiveness of the surface complexation and/or ion exchange processes as well as the electrostatic interaction in the arsenate adsorption on the composites.

### Adsorption isotherms

The isotherms of As(V) adsorption on UiO-66-NDC/GO nano adsorbent are examined at pH 7.8 and 30 °C, which are shown to be the best conditions. At a temperature of 298 K, 10 mg of adsorbent is added to 100 mL of aqueous samples (pH = 3) of adsorbate, with varied arsenate concentrations ranging from 5 to 25 mg/L. As demonstrated in Fig. 10, the adsorption capabilities of UiO-66-NDC/GO increase as the amount of As(V) in the solution sample increases. Due to a greater concentration gradient, more arsenate molecules are available in the vicinity of the active sites on the surface of the UiO-66-NDC/GO particles, resulting in this expected trend (the driving force of mass transfer).

The following linearized Langmuir, Freundlich and Temkin isotherms are used to analyze the data:

4 $$\frac{1}{{\mathrm{q}}_{\mathrm{e}}}=\frac{1}{{\mathrm{k}}_{\mathrm{L}}{\mathrm{q}}_{\mathrm{max}}}\cdot \frac{1}{{\mathrm{C}}_{\mathrm{e}}}+\frac{1}{\mathrm{qmax}}$$

5 $$\mathrm{qe}={K}_{f}+\mathrm{Ce}\frac{1}{n}$$

6 $${\text{q}}_{{\text{e}}} = {\text{BlnA}}_{{\text{t}}} + {\text{BlnC}}_{{\text{e}}}$$

where, qe—adsorption capacity (mg/g) at equilibrium, Ce -As(V) equilibrium concentration (mg/L), q_max_ is the maximum adsorption capacity, B = heat constant (J/mol), AT = Temkin constant, and T = temperature at 298 K, K_L_—equilibrium constant (L mg^−1^) representing the binding strength, and n, KF (mg/L) are the Freundlich constants specifying the adsorption and intensity capacity, respectively.

**Figure 12:**
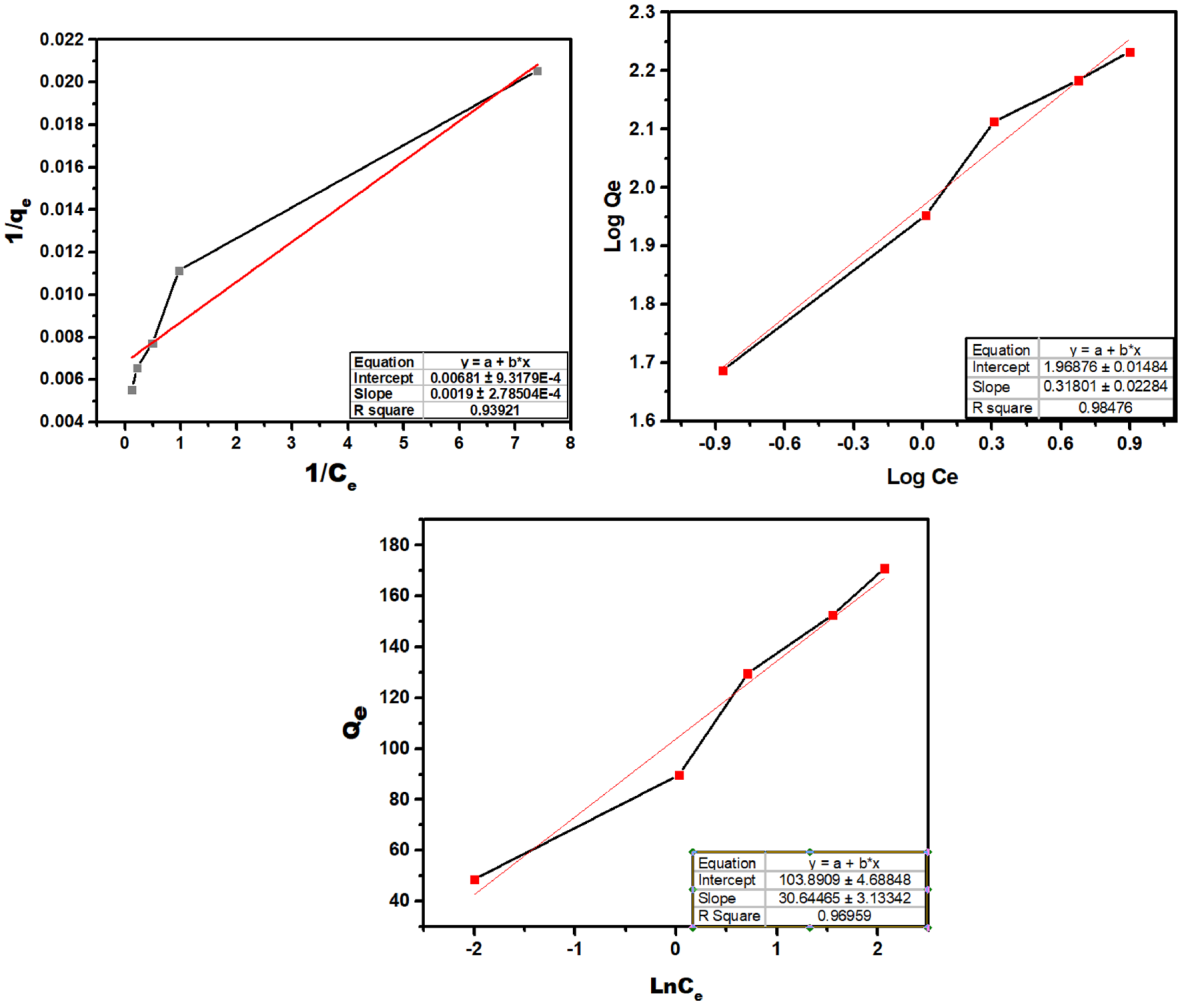
As(V) Adsorption isotherms of UiO-66-NDC/GO at 298 K., Good R^2^ values to adsorption data were obtained successfully obtained using linear fits of the Langmuir, Temkin, and Freundlich models.

Experimental data for UiO66-NDC-GO nano adsorbent is well fitted with all the isotherms as plotted in the Fig. 12 and the different parameters are represented in Table 2

**Table 2:** Different adsorption isotherms for adsorption of As(V) onto UiO-66-NDC/GO.

Intercept	Slope	q_max_ (mg/g)	KL	RL	R^2^
**Langmuir isotherm**					
0.00680	0.0019	**147.06**	3.58	0.01	0.939

Intercept	Slope	1/n	K_f_	R^2^	
**Freundlich isotherm**					
1.96876	0.31801	0.31801	93.05935	0.984	

Intercept	Slope	BT	KT	R^2^	
**Temkin isotherm**					
103.8909	30.64	30.64	29.68658	0.969	

Based on the criteria of the correlation coefficient, the maximum adsorption capacity using Langmuir isotherms was found to be 147.06 mg/g. A prior investigation utilizing Zn-MOF-74 for As(V) adsorption found a close match to the maximal capacity (99 mg/g). Our nano adsorbent removes almost one and a half time As(V) from simulated water under lab conditions. UiO-66-NDC/GO has a higher adsorption capacity than previously reported adsorbents for removing As(V), including 48.7 mg/g in HTZn-MOF-74, 99.0 mg/g in RT-Zn-MOF-74^25^ and 45 mg/g in zirconium oxide^26^. These findings highlight the significance of controlling particle size in adsorption processes. The results obtained from all characterization studies of composites performed after arsenate adsorption suggest that surface complexation and/or ion exchange processes are effective mechanisms in arsenate adsorption on the surface of the composites.

### Thermodynamic analysis

Adsorption data at three different temperatures are used to find the thermodynamic parameters of As(V) adsorption, such as variations in the standard enthalpy (H°), standard free Gibbs energy (G°), and standard entropy (S°), to provide a better description for the mechanism and nature of As(V) adsorption onto the UiO-66-NDC/GO surface. The thermodynamic nature of As(V) removal by UiO-66-NDC/GO is determined by calculating the values using Van’t Hoff equation at 298, 303, 308, and 313 K.

$$\mathrm{ln}\left(K\right)= \left(\frac{\Delta {H}^{o}}{R}\right)\frac{1}{T}+ \frac{\Delta {S}^{o}}{R}$$

where K = qe/Ce, ∆H° = Change in enthalpy, T = temperature, (∆G° = ∆H° − T∆S).

The positive value of H° corroborated the endothermic nature of arsenate adsorption on UiO-66-NDC/GO. ∆H° value of 0.80 kJ/mol^−1^ is likely due to chemisorption, which involves the dehydration of the metal atom and its surroundings (Table 3). As a result, the stated endothermic process is attributable to the arsenate anion, which gets more favoured as the temperature rises, replacing numerous water molecules. Furthermore, a positive value of S° suggests that the degree of freedom of the adsorbed arsenate increases with increased randomization at the solid/solution interface, implying that the stated dehydration happens mostly at the pore region and apart from the Zirconium cluster of the nanocomposite. The ΔH° and ΔS° values are calculated from the intercept and slope of 1/T versus lnkL. It is observed that the degree of the spontaneity of the adsorption process for As(V) decreased and increased in temperature. The adsorption process for As(V) using UiO-66-NDC/GO is physical as the value of enthalpy is less than 40 kJ mol^−1^. A positive value of entropy represents the random collision of As(V) species across the surface of the UiO-66-NDC/GO nanocomposite.

**Table 3:** Thermodynamic behaviour of UiO-66-NDC/GO for adsorption of As(V).

Temperature (K)	∆G°	∆S°	∆H°	R^2^
298.15	− 14.5897	51.63468	0.806552	**0.9916**
303.15	− 14.8446			
308.15	− 15.1041			
313.15	− 15.3639			

Significant values are in bold.

### Comparison studies of As(V) adsorption with previously reported adsorbents

As indicated in Table 4 below, different researchers have explored As(V) adsorption by various adsorbents. In comparison, novel synthesized UiO-66-NDC/GO is found to be a highly effective adsorbent for the adsorption of As(V) ions from water. The GO functionalized UiO-66-NDC have been proven to be a cost-effective alternative adsorbent for the removal of hazardous metals, particularly As(V) effluents, before they are discharged into the environment in this study.

**Table 4:** Comparison of UiO-66-NDC/GO for As(V) adsorption with previously reported studies.

Material/nano-composite	Adsorption capacity (mg/g)	pH	Surface area (m^2^/g)	Dose	Temperature (K)	References
UiO-66-NDC/GO	147.06	3	279.77	0.1 g L^−1^	298.15	This work
CCBB	26.13	6.7	38.27	–	298.15	^27^
MCBB	79.49	6.7	52.48	1.5 g L^−1^	298.15	^27^
*Turbinaria vulgaris* sp.	25.64	4.41	–	0.3 g L^−1^	298.32	^28^
Egg shell	8.43	4.1	7.91 ± 0.49	1 g L^−1^	293.15	^29^
PEI-coated bacterial biosorbent	62.9	4.0	–	3.0 g L^−1^	293.15	^30^
MnO_2_ impregnated alginate beads	6.5	6.5	2.04 ± 0.002	10 g L^−1^	298.15	^31^
Tea waste	4.92	7.0	4.03 ± 0.61	1 g L^−1^	293.15	^29^
IMIGAC	16.0	4.0	420.12	3.33 g L^−1^	293 K	^32^
RGO-MFT	77.6	6.0	275.23	0.2 g L^−1^	303.15	^33^
Fe-TNTs	36.41	2.5	162.8	0.2 g L^−1^	298.15	^34^
TiO_2_-Fe_2_O_3_ bi-composite	12.4	5.0	133.5	–	298.15	^35^
	7.79	7.0				
	6.48	9.0				
Ce-Ti oxide	7.5	6.5	38.2–68.8	–	298.15	^36^
NHITO	14.3	7.0	77.8	2.0 g L^−1^	303.15	^37^
m-TiO_2_-αFe_2_O_3_	99%	7.0	95	–	298.15	^38^

### Desorption experiment of As(V) loaded on the UiO-66-NDC/GO

**Figure 13:**
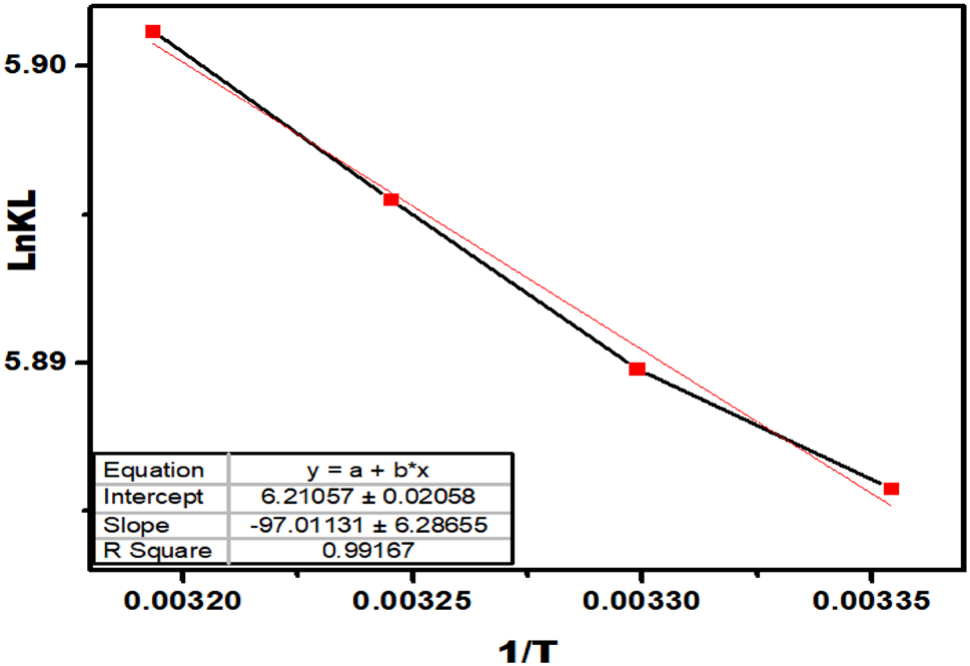
As(V) adsorption isotherms of UiO-66-NDC/GO as a function of temperature.

**Figure 14:**
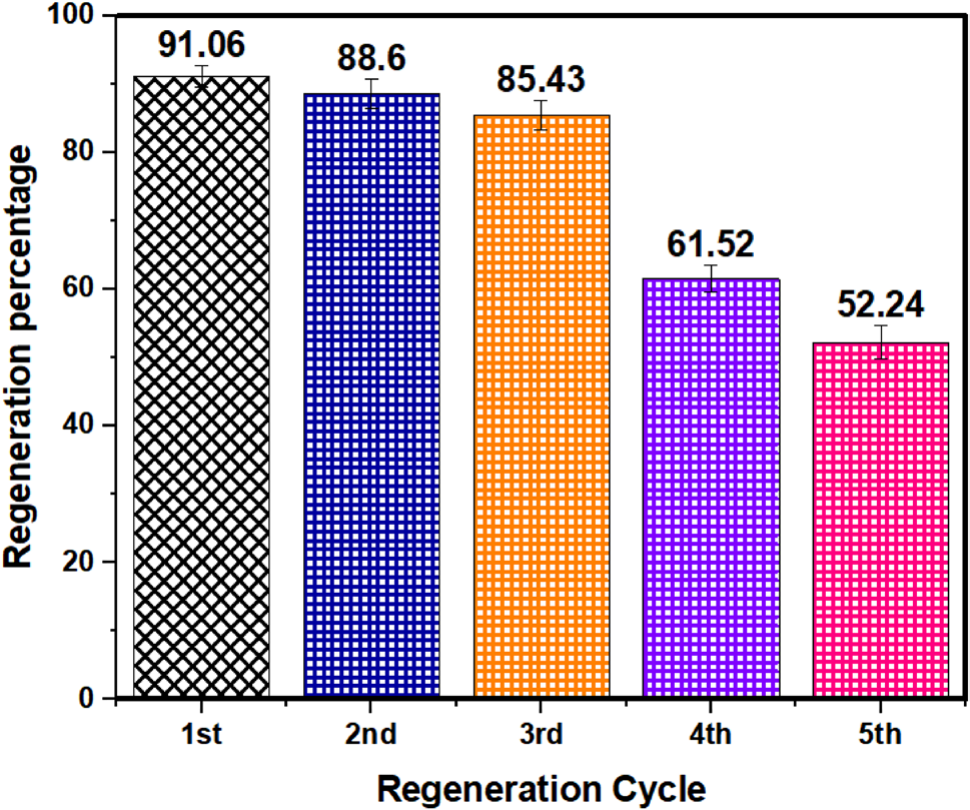
Regeneration and reusability UiO-66-NDC/GO for As(V) removal.

UiO-66-NDC/GO desorption-regeneration tests were carried out to check the reuse feasibility and recovery. The removal efficiencies of UiO-66-NDC/GO following six cycles of desorption-regeneration with 0.1 M HCl solution are shown in Fig. 13. After three regenerations, the removal effectiveness of MOF had dropped somewhat from 91.06 to 88.6%, respectively. After five cycles, their removal efficiency was only about 52.24% of what it was at the start, indicating that UiO-66-NDC/GO may have a potential recycling property for As(V) removal as shown in Fig. 14.

## Conclusion

In this study, UiO-66-NDC/GO was reported for the first time as the adsorbent for the removal of As(V) from water. This is the first time UiO-66-NDC/GO has been used to remove arsenic from water, to our knowledge. The UiO-66-NDC/GO performed well across a wide pH range, from extremely acidic 1 to basic 10, with the best adsorption efficacy at pH 3. At the ideal pH, the UiO-66-NDC/GO adsorbent had a remarkable As(V) uptake capacity of 147.06 mg/g. This is the highest recorded arsenate adsorption capacity, far above other synthetic and commercial adsorbents. All UiO-66-NDC/GO characterization investigations conducted after arsenate adsorption indicate that surface complexation and/or ion exchange processes are effective mechanisms in arsenate adsorption on the UiO-66-NDC/GO surface.

Interestingly, the theoretical simulation calculations confirm the dynamic superiority of UiO-66-NDC/GO nanocomposite with finite values of delocalized Zr atoms over the surface compared to the GO system making the nanocomposite system a relevant source of As adsorption. This simulation output is fully supporting the discussed experimental results for in-depth understanding. To summarise, this research adds to our understanding of the use of UiO-66-NDC/GO in water treatment. The extremely porous structure incorporating zirconium oxide clusters provided a greater contact area and more active sites in unit space, allowing UiO-66-NDC/GO adsorbent to have a higher adsorption capacity than most typical nanoparticle adsorbents. UiO-66-NDC/GO could be a promising advanced adsorbent in the arsenic clean-up business due to its high adsorption efficiency toward aquatic arsenic species.

## Methodology

### Theoretical first principle-based simulation details

Theoretically, the pristine graphene oxide (GO) and UiO-66-NDC/GO nanocomposite systems are considered for the first principle-based density functional simulation studies. Primary geometry optimization with total energy values has been performed with the Quantum Espresso softwares^39^ within generalized gradient approximation (GGA) following Perdew-Burke-Ernzerhof (PBE) format^40^. Van der Waals dispersion correction (vdW-DF) has been implemented for both systems to get accurate results throughout the calculations. Specific k-point grids of 9 × 9 × 1 have been considered here at plane wave cutoff energy of 540 Ry with a force of less than 0.001 eV/Å to get optimized structure. It is necessary to fix a vacuum region of 20 Å along the z-direction to minimize the periodic image interaction during the simulation.

### Reagents

All chemicals, reagents, and salts were of the highest purity and were used without any purification. Graphite powder (< 20 μm Sigma-Aldrich), Zirconium (IV) chloride (99.95% STREM), Potassium permanganate (≥ 99.0% Sigma-Aldrich), and 1,4-Napthalenedicarboxylic acid (> 95% TCI). 1,4-Napthalenedicarboxylic acid was used as an organic linker and Zirconium (IV) chloride as a precursor. N,N-Dimethylformamide (99.8% Sigma Aldrich) was used as a solvent to dissolve the reactants. Sodium arsenate (Na_2_HAsO_4_.7H_2_O 99%, SDFCL) was used for the preparation of the stock solution, in which a specific amount of Na_2_HAsO_4_.7H_2_O was dissolved in pure water. The stock solution was then diluted in deionized water for the preparation of various batches for adsorption studies.

### Synthesis procedure for graphene oxide (GO)

GO was synthesized using the improved hummers method in which graphite powder and KMnO_4_ (1:6 ratio) were dispersed in a mixture of phosphoric acid and sulfuric acid (1:9 ratio) for 12 h at 65 °C. After that, the reaction mixture was allowed to cool, and 400 mL of ice-cold water was added along with 5 mL of hydrogen peroxide. Then allow the mixture to stir for half an hour. centrifuge the reaction mixture for 6 min at 8000 rpm. The product material was washed with a succession of deionized water, ethanol, HCl, and finally diethyl ether. The resultant was dried at room temperature in an air-controlled vacuum chamber for 6 h^41^.

### Synthesis of UiO-66-NDC/GO

UiO-66-NDC/GO was prepared using a solvothermal procedure. 0.5 wt.% of graphene oxide was sonicated for 8 h in DMF and then mixed with zirconium chloride (0.005 mol and left on a magnetic stirrer for 12 h. after that, add 0.005 mol of 1,4-naphthalene dicarboxylic acid to the mixture solution and mix it completely. Pour the mixture into a Teflon liner within a stainless-steel autoclave (200 mL), and the solution was heated to 120 °C for 24 h. after 24 h, allow the material to cool, centrifuge the reaction mixture at 5000 rpm for 10 min, and wash 3 to 4 times with DMF and ethanol. The precipitate was collected and washed several times with DMF and ethanol to ensure the purity of the synthesized nano adsorbent.

### Characterization

Morphological and structural characteristics of the nano-adsorbent were studied using Raman, UV, FTIR, SEM–EDS, XRD, and BET techniques. Raman characterization of the synthesized material was recorded on STR-300 confocal Raman spectrometer (Seki Technotron Corp., Japan), with diode laser excitation at 785 nm. The data deconvolution of Raman was done by GRAMS/AI data processing software. UV spectrum was performed on a Perkin Elmer (Lambda 35) ranging from 200 to 800 nm at a sweep rate of 480 nm/min. TEM of the UiO-66-NDC/GO before and after the experiment was recorded on Titan Themis (Thermofisher) operated at 300 kV. The UiO-66-NDC/GO were grounded into a fine powder using pestle mortar and then mixed with ethanol for dispersion. An ultrasonic bath was used for further dispersion processes. For observational purposes, a drop of the suspension was dropped onto a typical copper grid with carbon coating. Functional groups of the synthesized material were recorded on a Bruker spectrometer (TENSOR II) in ATR mode from 400 to 4000 cm^−1^ of the spectral region at a resolution of 4 cm^−1^. Composition and Surface morphology was carried out using SEM–EDS on Zeiss ULTRA 55. XRD analysis of the nano-adsorbent was examined on a Bruker D8 Advance X-ray diffractometer at a scan speed of 6°/min from with 2θ ranging from 5° to 80°. Surface area and pore size distribution were measured using Micromeritics ASAP 2020. The sample was degassed for 150 min overnight, and calculations were made. All the structural and morphological characterization of the UiO-66-NDC/GO were recorded in the premises of the IISc Bangalore.

### Surface charge of nano-adsorbent

The point of zero charges for nano-adsorbent was determined by following the salt addition method 0.1 M of NaNO_3_ in 250 mL flasks was attuned to various pH of 2–11 (pH_initial_) using 0.1 M NaOH and 0.1 M HCl. 100 mg of the adsorbent were added to all the flasks and kept at 180 rpm in a shaker at room temperature. The pH of all the flasks was measured after 24 h (pH_final_) using a pH meter, and the obtained readings were plotted against the initial pH (pH_initial_). The initial pH at which change in pH is zero exhibits a point of zero charges^42^.

### Adsorption experiments for As(V)

As(V) removal by adsorption using nano-adsorbent was carried out using batch adsorption studies. All As(V) adsorption experiments were performed at 25 °C, excluding the thermodynamics studies. The equilibrium capacity for As(V) was calculated using

7 $$q= \frac{{(C}_{t }-C) V }{m}$$

where C_t_—initial As(V) concentration, C—equilibrium As(V) concentration, m = mass of nano-adsorbent, V—the volume of As(V).

For adsorption studies, a stock solution of 10 mM of As(V) by mixing a known quantity of Na_2_HAsO_4_.7H_2_O in a 500 ml conical flask for further experimentation. For studying pH effect on adsorption, solutions with different pH (2–11) were mixed with 0.1 g/L of nano-adsorbent in an incubator for 3 h. Different parameters for optimization, such as adsorbent dose and initial ion concentration, were carried out by changing dose and concentration at fixed pH. Each flask was filtered using a 0.22 um syringe filter and analysis was done on Thermo X Series II, and the data was taken in replicates to ensure a standard error of less than 1% (Supplementary information S1).

## Supplementary Information

Supplementary Figures.

## Data Availability

The data that support the findings of this study are available from [Praveen C Ramamurthy]. Still, restrictions apply to the availability of these data, which were used under license for the current study, and so are not publicly available. However, data are available from the authors upon reasonable request and with permission of [Praveen C Ramamurthy].
