# Supplementary material for: A systematic study of arsenic adsorption and removal from aqueous environments using novel graphene oxide functionalized UiO-66-NDC nanocomposites
Source: Sci Rep. 2022 Sep 22;12:15802. doi: 10.1038/s41598-022-18959-2 (PMC9500003; doi:10.1038/s41598-022-18959-2)
Supplement: Supplementary file 1 — Supplementary Figures. [file 41598_2022_18959_MOESM1_ESM.docx]

**Supplementary Data**

**Characterization of graphene oxide**


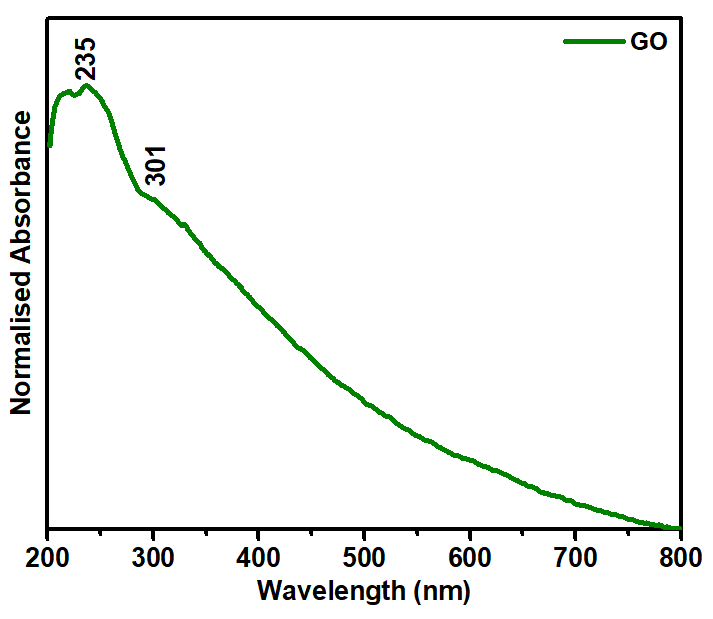


**Supplementary Fig. 1 shows the UV pattern of graphene oxide.**

UV spectra at 235 nm are assigned to π-π∗ transitions of aromatic C–C ring in graphene oxide. The results are similar to the previous finding of many researchers (Johra et al. 2014; Singh et al. 2022).A shoulder peak around 301 nm was also observed which is assigned to the n → π∗ transition of the C=O groups as shown in the Supplementary Fig 1.(Marcano et al. 2010).

**XRD Data:**


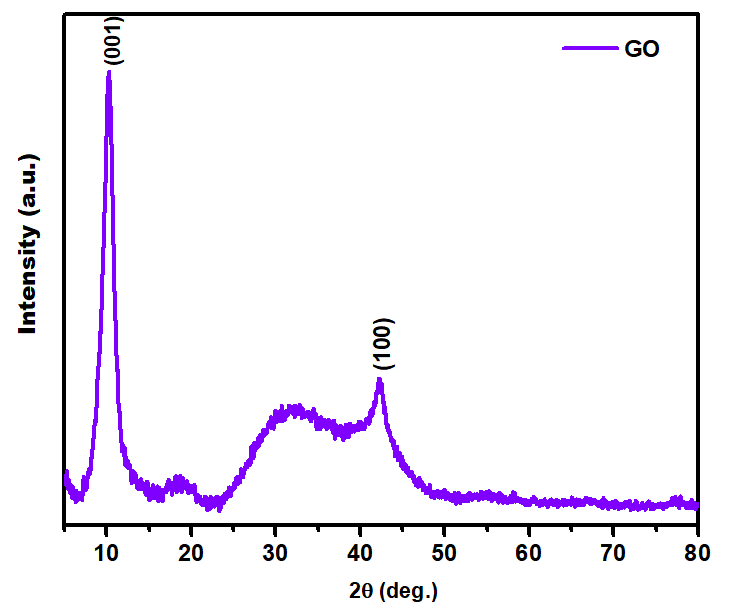


**Supplementary Fig-2: XRD data of graphene oxide**

The as synthesized GO material was characterized using XRD, as shown in Supplementary Fig-2. The diffraction peak at 2θ of 10.26 and 42.36 from (001) and (100) planes, respectively. The peak at 2θ = 10.26 with d-spacing of 8.9 A which was indicated as a characteristic peak of GO (Marcano et al. 2010). XRD data shows that we have successfully synthesized GO material.

**FTIR data:**


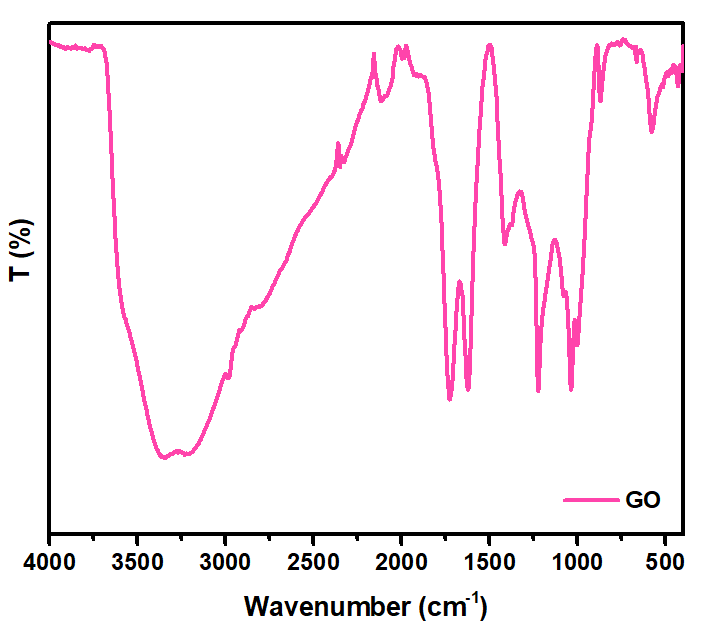


**Supplementary Fig-3: FTIR spectrum of graphene oxide.**

The vibrational frequencies of GO material are characterized by FTIR, as shown in Supplementary Fig-3. The strong and broad peak around 3300 cm^-1^ and 1624 cm^-1^ is related –OH functional group stretching and bending vibration, reveals the presence of water molecule adsorbed on the GO material. The peaks at 1727 cm^-1^ is attributed to C=O stretching vibrations. The peak at 1225 cm^-1^ is attributed to C-O-C stretching vibration and peak at 1034 cm^-1^ is attributed to C-O vibrations. The peak at 1415 cm^-1^ is attributed to C-H bending vibrations of –CH_3_ group. The presence of C=O and C-O functional groups in GO material further confirmed that the graphite is oxidized (Marcano et al. 2010; Singh et al. 2022).
